# Effect of Waste Glass as Fine Aggregate on Properties of Mortar

**DOI:** 10.3390/ma15238499

**Published:** 2022-11-29

**Authors:** Wei Chen, Song Dong, Yuehan Liu, Yue Liang, Frederic Skoczylas

**Affiliations:** 1 School of Civil Engineering, Architecture and Environment, Hubei University of Technology, Wuhan 430068, China; 2 CNRS—Centre National de la Recherche Scientifique, Centrale Lille, UMR9013—LaMcube—Laboratoire de Mécanique Multiphysique et Multiéchelle, Université de Lille, F-59000 Lille, France

**Keywords:** waste glass sand, replacement, gas permeability, compressive strength, pore structure

## Abstract

Currently, most cities landfill most waste glass, resulting in the waste of resources and environmental pollution. Therefore, to realize the recycling of waste glass, solid waste glass was recycled and broken. Waste glass sand was prepared according to the gradation of natural river sand particles and the fineness modulus screening. It was used as an alternative material to natural river sand and mixed with mortar materials with different replacements. Analysis of the mortar with different replacements (0%, 20%, 40%, 60%, 80%) was conducted by combining macro and micro tests on the change law and influence mechanism of permeability, mechanical properties, and microstructure. The results showed that: the replacement of waste glass sand effectively improved the gas permeation resistance of mortar; with the increase of replacement, the gas permeation resistance of mortar roughly showed a trend of increasing first and then decreasing. The replacement of waste glass sand at 20% can better promote cement’s hydration so that the mortar’s porosity is reduced by 16.5%. The gas permeability decreases by 57.4%; the compressive strength increases by 3%, and the elastic modulus increases by 5.9%. When the replacement rate of glass sand is 20%, the test performance of mortar is the best among the five groups.

## 1. Introduction

With the rapid development of the construction industry, natural river sand, the raw construction material, has been mined in large quantities. While river sand is a non-renewable resource, the increase in mining has led to the existing natural river sand being in a resource shortage. In recent years, the supply of natural river sand has been tight; so, to reduce the cost of construction, it is urgent to find an alternative to the raw construction material of sand and gravel [1,2]. We use glass widely in our daily life, such as flat glass, glass bottles, glassware, vacuum tubes, and other products. Glass is available as an ideal material for recycling. Currently, most of the waste glass in China is disposed of by the landfill method, which causes environmental pollution and the waste of resources [3,4,5]. Therefore, to realize waste glass’s resource utilization, its application in building material mortar and concrete is now being studied [6,7].

The current research by related scholars on waste glass granular concrete was carried out in four forms: coarse aggregate, fine aggregate, waste glass fiber, and waste glass powder as single or compound substitution [8,9,10,11,12]. Guo [13] studied the permeability, density, consistency change patterns, and related effects of construction mortars with different blends of waste glass sand. Affiliated scholars chose broken glass sources such as waste lamps or city glass and soda lime glass to partially replace natural sand or cement to study the thermophysical properties of green mortars. The results showed that adding waste glass powder to ordinary cement mortars could significantly affect their thermophysical properties [14,15]. Some studies found that mortars containing waste glass can be a viable filler material in radioactive waste disposal facilities [16]. Zhang [17] proposed a new research method to mix waste glass material into alkali-excited cement-based slurry or mortar. Moreover, experimental results showed that ASR expansion could be further reduced by adjusting the crushed glass (GC) ratio to the binder. Several scholars have investigated the feasibility of waste glass as fine aggregate, coarse aggregate, and powder with different waste glass replacement mortars or concrete [18,19,20].

Studies related to the compressive strength, modulus of elasticity, and splitting tensile strength of mortar were carried out after replacing natural sand with waste glass. The results showed that the addition of waste glass sand aggregate improved the mechanical properties of mortar [21,22]. Many scholars have conducted experimental studies on fluidity, shrinkage, strength, pore size distribution, and scanning electron microscopy after incorporating waste glass into mortar or concrete materials to establish the relationship between macroscopic properties and microstructure [23,24,25]. Khan [26] used waste glass flakes to study the properties of alkali-activated fly ash and GGBFS mixed mortars. The study showed that using different proportions of waste glass particles in alkali-activated mortars to replace natural river sand had comparable effects to using natural river sand.

Using recycled waste glass as a partial replacement for natural river sand in concrete, the researchers conducted a series of tests to determine the composition and durability properties of the concrete to investigate the suitability of the waste glass particles in concrete. The test results showed that adding waste glass particles reduced the swelling caused by the alkali–silica reaction [27,28,29]. The permeability of cement-based materials has been a hot research topic in civil engineering materials, and permeability is one of the critical factors in durability problems. Many scholars have studied the effect of chloride ion permeation corrosion on cement-based materials [30,31,32]. Alireza studied the substitution of crushed rubber for sand and used nano-silica and metakaolin admixtures to compensate for strength degradation and improve the workability of concrete. Fibers were used in concrete mixes to improve the mechanical properties and strength and limit crack extension in concrete. It improves the wear resistance of concrete [33,34,35]. The operational performance of different commercial cement types under different temperature and humidity conditions has been studied to investigate the concrete durability factors [36,37].

In view of the fact that the related scholars currently studying the replacement of natural river sand by waste glass particles have not formulated it according to the particle gradation of natural river sand, which will affect the overall effect of waste glass particles when replacing natural river sand to a certain extent. Gas permeability can be used as an index to reflect the durability performance of cementitious materials. The research on the gas permeability of waste glass particles in cementitious materials is still insufficient.

This study prepares waste glass sand according to the same granularity and fineness modulus of waste glass particles as natural river sand. The waste glass sand is closer to natural river sand in terms of apparent properties, and waste glass mortar is prepared with different doping amounts. Gas permeation, mechanical, nuclear magnetic resonance, scanning electron microscope, and XRD were carried out. The optimum substitution rate of waste glass was studied, making the performance of mortar materials achieve the best result. This study provides a theoretical basis for the resource utilization of waste glass in cement-based materials.

## 2. Materials and Methods

### 2.1. Raw Materials and Specimen Preparation

**Figure 1:**
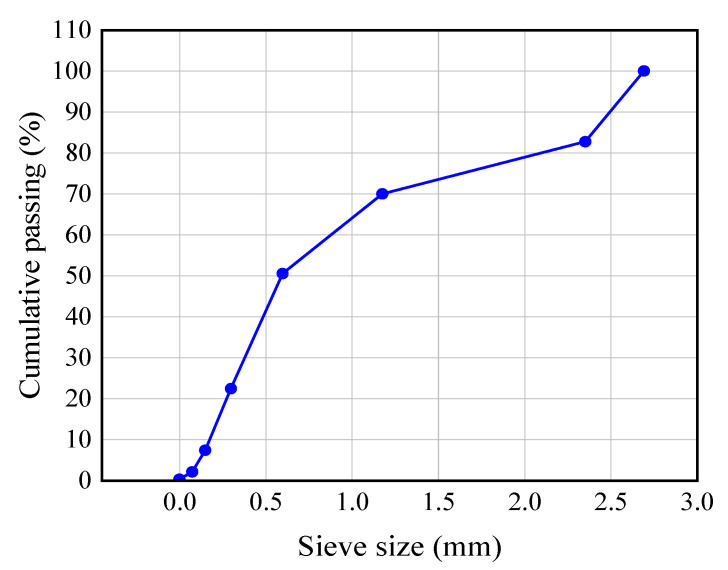
Grading curve of natural river sand.

The raw materials included cementitious material P·O 42.5 ordinary silicate cement; the fine aggregates were natural river sand and waste glass sand. The fineness modulus of natural river sand is 2.7; the apparent density of natural river sand is 2.58 g/cm^3^, and the bulk density is 1.48 × 10^3^ kg/m^3^. The particle size distribution of natural river sand is shown in Figure 1.

**Figure 2:**
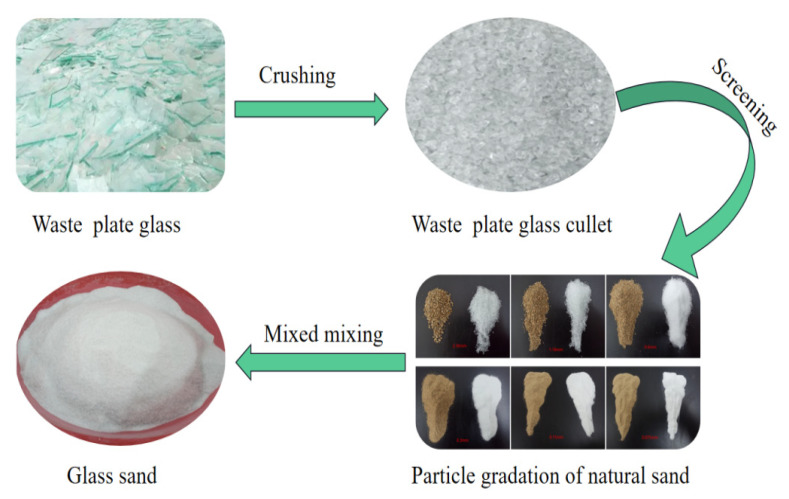
Waste glass sand production process.

The waste glass particles used in the test were obtained by processing colorless and transparent flat glass, which has a uniform texture and irregular shape, to be closer to the apparent properties of natural river sand. The waste glass sand was prepared by sieving and mixing the waste glass particles according to natural river sand’s particle distribution and fineness modulus. The waste glass sand preparation process is shown in Figure 2.

The mix proportion is shown in Table 1; the amount of waste glass sand substituted for natural river sand, cylindrical mortar specimens of Φ50 mm × 100 mm were prepared with a water–cement ratio of 0.5. The waste glass sand replacement was approximately 0%, 20%, 40%, 60%, and 80% of the mass of the total aggregate (natural river sand). The specimens were grouped as B0, B20, B40, B60, and B80. The specimens were demolded 24 h after the pouring and vibrating. Specimens were stored in water at a temperature of 20 ± 2 °C for 60 days. The two ends were polished with a double-sided end grinder, and the specimens were put into the vacuum saturation apparatus for re-saturation treatment. Dry material state is taken after oven-heating at 60 °C until the mass change is less than 0.1%.

### 2.2. Testing Methods

#### 2.2.1. Gas Permeability Test

**Figure 3:**
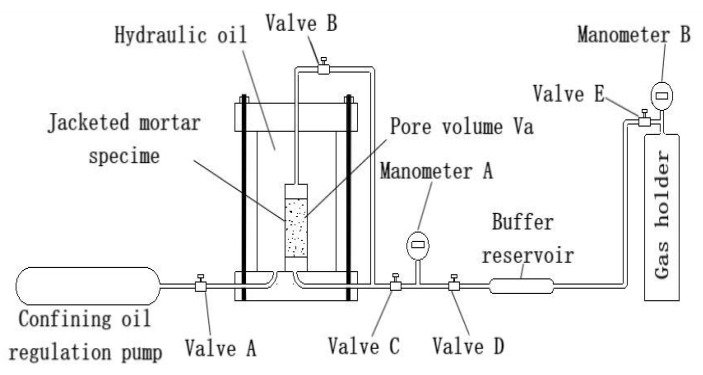
Schematic diagram of gas permeation test device.

The test device used was a gas–liquid permeation system (Figure 3). It is mainly composed of gas injection system, confining pressure chamber and HDH100-4 servo machine. The limit gas permeability measured by the device is 10^−22^ m^2^, and the maximum confining pressure is 60 MPa [38,39].

Gas permeability measurements were performed during loading and unloading confining pressure cycles (3, 5, 10, 15, 20 MPa). The average flow rate at the inlet end is calculated using Equation (1):

(1) $$Q_{v}=\frac{V_{r}\Delta P}{P_{m}\Delta t}$$

where *Q*_v_ is the average flow rate at the inlet end (m^3^/s); *V_r_* is the buffer reservoir volume; ∆*P* is the difference between the initial pressure and the final pressure at the inlet end; *P*_m_ is the average pressure at the inlet end (MPa); ∆t is the time variation difference.

Gas permeability *K* along specimen height h is given by Equation (2):

(2) $$K=\frac{2\mu hQ_{v}P_{a}}{A({P_{m}}^{2}-{P_{a}}^{2})\Delta t}$$

where *K* is the gas permeability coefficient (m^2^); *μ* is the argon viscosity coefficient (Pa·s); h is the height of the specimen (mm); *A* is the cross-sectional area of the specimen (m^2^); P_a_ is the pressure value at the outlet end (0.1 MPa).

#### 2.2.2. Compressive Strength Test

The loading system is the ETM series electronic universal testing machine with a maximum loading capacity of 300 kN. The axial displacement rate was 0.1 mm/min for all tests. The data acquisition system measures the loading force and the displacement of the test machine.

#### 2.2.3. Nuclear Magnetic Resonance Test

The instrument used in this test was Newmark’s NMR analyzer (PQ-001, Manhattan, NY, USA). Before the start of the NMR test, the specimens were vacuum-saturated to fill the internal pores of the mortar with complimentary water. The mass of the saturated specimens was measured using a precision balance. The NMR relaxation time method is based on the principle that the signal intensity of hydrogen atoms in the vacuum-saturated specimen is detected to provide feedback on the pore structure inside the specimen. The NMR relaxation time is divided into longitudinal relaxation time T_1_ and transverse relaxation time *T*_2_, and the transverse relaxation time *T*_2_ is more sensitive to the pore size, both of which satisfy Equation (3):

(3) $$\frac{1}{T_{2}}=\rho \frac{S}{V}$$

where *ρ* is the relaxation strength of the test material, and its value is related to the material category; *S* is the surface area of the specimen pore; *V* is the pore volume of the specimen.

#### 2.2.4. X-ray Diffraction Test

XRD was tested by a Rigaku Ultima IV X-ray diffractometer (Tokyo, Janpan). The mortar specimens were ground, sieved with a 320 mesh sieve, and dried for storage. The 2θ scanning range was 5~80°; the voltage was 40 kV; the scanning current was 40 mA, and the scanning speed was 2°/min.

#### 2.2.5. Scanning Electron Microscope Test

A SEM test was performed using a ZEISS Gemini SEM 500 field-emission scanning electron microscope from Carl Zeiss (Jena, Germany) to observe and analyze the internal microscopic morphology of the dried mortar. The main parameters of the instrument are: storage resolution up to 32 k × 24 k pixels; acceleration voltage: 0.02–30 kV; magnification: 50×–20 k×; probe current: 3 pA–20 nA.

## 3. Results and Discussion

### 3.1. Gas Permeability

#### 3.1.1. Initial Gas Permeability and Porosity

**Figure 4:**
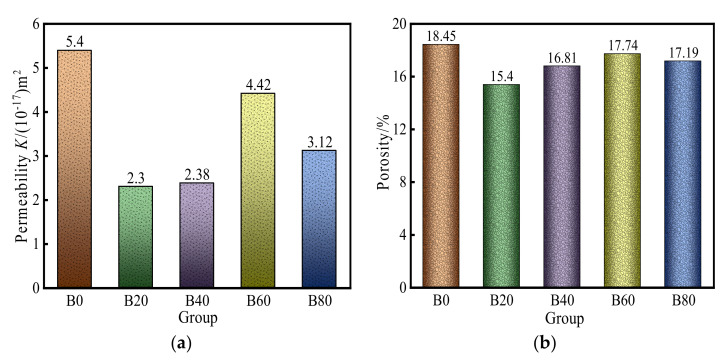
Initial gas permeability and porosity measured: (**a**) gas permeability, (**b**) porosity measured.

Initial gas permeability at 3 MPa confinement and porosity are shown in Figure 4a,b. As shown in Figure 4a, the replacement of waste glass sand significantly reduced the gas permeability of mortar. Compared with the control group, the initial gas permeability of mortar with different waste glass sand replacements decreased by 57.4%, 55.9%, 18.1%, and 42.2%, respectively. The mortar specimens with 20% waste glass sand replacement had the lowest gas permeability, indicating that the small replacement of waste glass particles made the mortar pore change significantly. Figure 4b shows the mortar porosity measured by the water-loss method, and the porosity of mortar with different replacements was reduced by 16.5%, 8.9%, 3.8%, and 6.8%, respectively, compared with the control mortar. The results indicated that the replacement of waste glass sand reduced the number of pores inside the mortar and made the internal pores more compact.

#### 3.1.2. Effect of Confining Pressure on Gas Permeability

**Figure 5:**
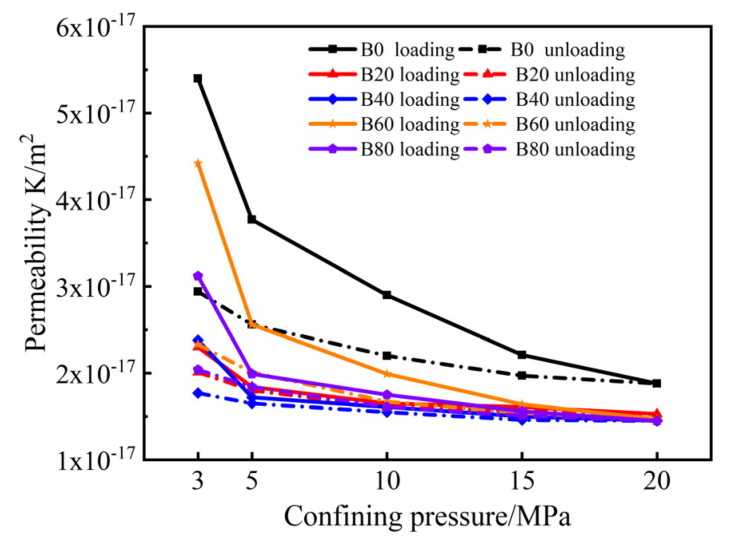
Variation of gas permeability with confining pressure.

The variation in the gas permeability of mortar with the confining pressure for each replacement is shown in Figure 5. The gas permeability of the specimens decreased by 30.2%, 20%, 27.7%, 42.3%, and 36.2% when the confining pressure loading from 3 MPa to 5 MPa. The gas permeability decreased by 23.1%, 10.3%, 11.4%, 22.3%, and 12.1% when the confining pressure loading from 5 MPa to 10 MPa. The gas permeability of the specimens decreased by 23.8%, 2.4%, 6.8%, 17.6%, and 10.9% when the confining pressure loading from 10 MPa to 15 MPa. The gas permeability decreased by 14.9%, 1.2%, 3.3%, 10.4%, and 7.1% when the confining pressure loading from 15 MPa to 20 MPa, respectively. It indicated that the rate of decrease in gas permeability for all specimens was the fastest when the confining pressure loading from 3 MPa to 5 MPa. At the initial loading of the confining pressure, the pores inside the specimen were the most relaxed, and there were more paths for gas permeation, so the gas permeability of the specimen at this stage decreased enormously. With the continuous loading of the confining pressure, the pores inside the specimen were squeezed, and the pores became narrowed and clogged. Furthermore, the gas permeability decreased more slowly when the confining pressure loading reached to a higher value, which indicated that a threshold value in the process of gas permeability changed with the confining pressure. The influence of confining pressure on the gas permeability was significant, and the gas permeability showed a gradually decreasing trend with the increase of confining pressure.

The test gas permeability is normalized in order to reduce the error of the test data, thus defining the dimensionless permeability γ as Equation (4):

(4) $$\gamma =\frac{K_{i}}{K_{3}}$$

where *K*_i_ is the gas permeability at all levels of envelope pressure, and *K*_3_ is the gas permeability measured at 3 MPa confining pressure.

In Table 2, the parameters of the fitted mortar are dimensionless permeability. b is the gas permeability confining the pressure-sensitivity coefficient, and the magnitude of the absolute value of b affects the trend of the fitted curve. The larger the absolute value of b, the more precipitous the fitted curve, and the greater the confining pressure sensitivity. Conversely, the fitted curve is flatter the smaller the confining pressure sensitivity.

**Figure 6:**
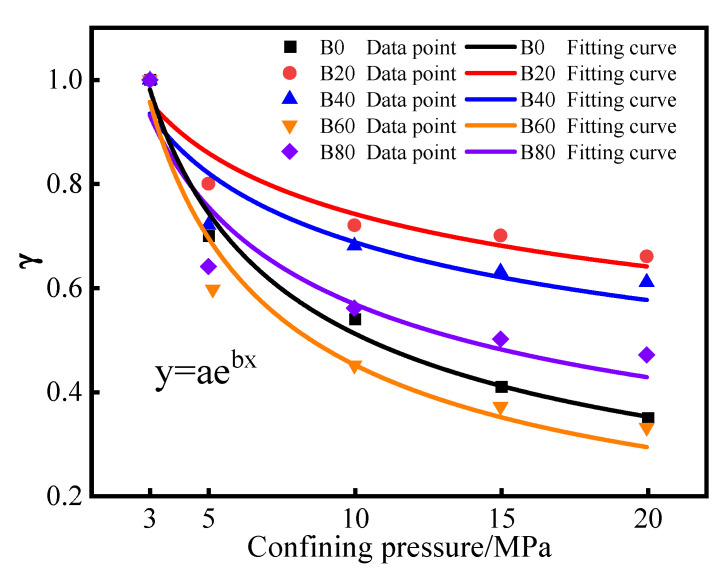
Confining pressure-sensitivity fitting curve.

Figure 6 shows a fitted curve of the confining pressure sensitivity, combined with the values of the fitted parameters in Table 2. The fitted correlation coefficient R_2_ is above 0.90, which shows that the gas permeability of the mortar with each replacement shows a good power function relationship with the confining pressure sensitivity. It indicates that the absolute value of the confining pressure-sensitivity coefficient b showed a trend of increasing and then decreasing with the increase of waste glass sand replacement. It can be concluded that the mortar under 20% waste glass sand replacing was subject to the least confining pressure sensitivity, so b can reflect the relationship between gas permeability and confining pressure sensitivity. It can also be seen from Figure 6 that, when the confining pressure loading from 3 MPa to 5 MPa, the dimensionless gas permeability of the specimens for all replacement decreased by 30%, 20%, 28%, 42%, and 36%. This indicated that the dimensionless permeability showed a trend of decreasing and then increasing. Moreover, the gap between the decreases in the dimensionless permeability of specimens became smaller and smaller. Because the confining pressure sensitivity of the specimens is in the range of 15–20 MPa, the effect of confining pressure on the internal pore structure of mortar specimens is relatively minimal. The specimens with 20% waste glass sand replacement were more capable of inhibiting the deterioration of the pore structure. Overall, the initial permeability at this replacement was the lowest, and the permeability decreased the slowest.

### 3.2. Mechanical Property Analysis

**Figure 7:**
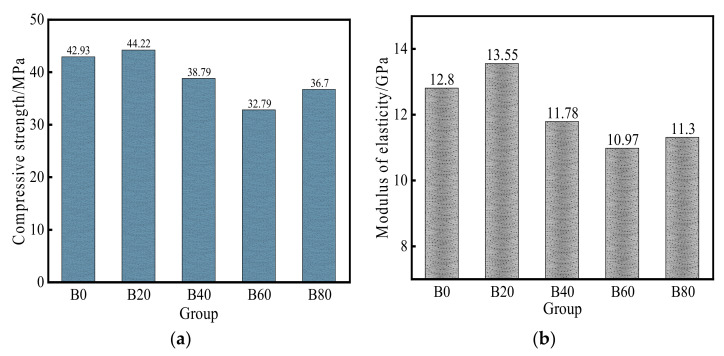
Compressive strength and elastic modulus of mortar for each replacement: (**a**) compressive strength; (**b**) elastic modulus.

The compressive strength of mortar is shown in Figure 7a, and it can be seen that specimen B20 had the highest compressive strength among the five groups of specimens, and the compressive strength was increased by 3% compared with the control group. The compressive strength of specimen B20 may be due to the waste glass sand being prepared according to the particle gradation of natural river sand when the waste glass sand was mixed with 20% replacement. The silica in a small number of fine glass particles reacted with the calcium hydroxide produced by the hydration of cement. It produced more C–S–H gel, which increased the impermeable pore density inside the specimen and increased the compressive strength of the mortar. This may also be due to the densification of the microstructure in the interstitial transition zone. The increase in strength was due to the volcanic ash activity of the fine glassy aggregate [39,40].

It can also be seen from Figure 7a that the compressive strength of B40, B60, and B80 specimens decreased by 9.6%, 23.6%, and 14.5%, respectively, compared with the control group, indicating that the strength of the mortar all decreased with the increase of waste glass sand replacement. It is mainly due to the gradual decrease of natural river sand and the gradual increase of waste glass sand; the angle of the fine glass aggregate may also increase the internal pores of mortar. While the surface of waste glass sand is relatively clean and smooth, the bond strength of cement after hardening is smaller when there is an external force acting on mortar [11,13,26]. It is easy to damage the interface, so the compressive strength of the mortar was reduced. The compressive strength of the B80 specimen was higher than that of the B60 specimen because more waste glass sand was mixed in, which made more waste glass sand with fine glass particles and a cement hydration reaction to produce more gel [16]. Thus, the compressive strength of the B80 specimen was higher than that of the B60.

**Figure 8:**
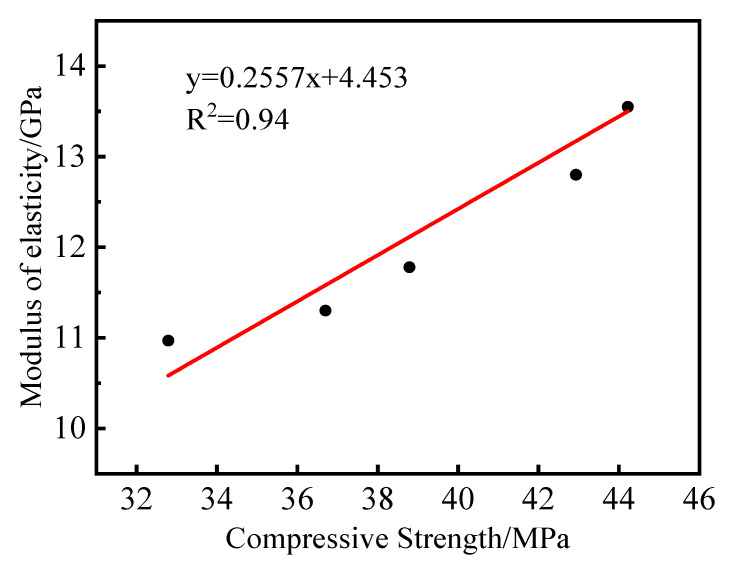
Linear fit of elastic modulus to compressive strength.

The elastic modulus of mortar is shown in Figure 7b, and it can be seen that the trend of the elastic modulus of mortar was approximately the same as that of the compressive strength. The elastic modulus of the B20 specimen was also the largest; its elastic modulus was 5.9% higher than that of B0, and the elastic modulus of B40, B60, and B80 was 7.8%, 14.3%, 18.4, and 11.7% lower, respectively, compared with B0. From a macroscopic point of view, the 20% replacement had the most vital ability to resist elastic deformation, which meant that this replacement mortar had the best effect in bonding cement paste together, and the angular size of the waste glass sand can be attributed to better interlocking between cement paste and waste glass sand. With the increase of waste glass sand replacement, the mortar elastic modulus was smaller than the control mortar because glass particles were brittle materials and more brittle than natural river sand [21,26]. Therefore, the increase of waste glass sand replacement led to mortar elastic modulus lower than natural mortar and lower than 20% replacement mortar. A linear fit of mortar modulus of elasticity to maximum compressive strength with R^2^ = 0.94 is shown in Figure 8, which can be observed to have a strong correlation.

**Figure 9:**
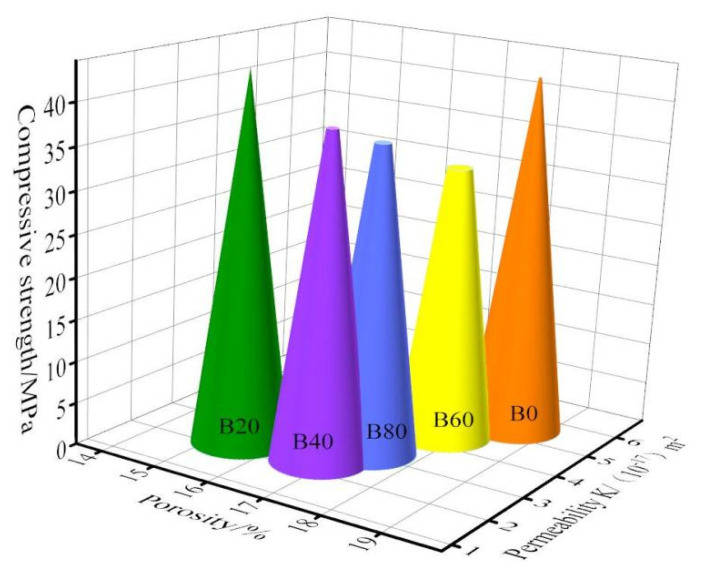
Relationship between porosity, gas permeability, and compressive strength.

Figure 9 shows the relationship between porosity, gas permeability, and compressive strength. The mortar porosity was the smallest, and the permeability was also the smallest when the replacement was 20%. The compressive strength of mortar under this replacement was the largest. At the same time, the glass incorporation reduced the mortar porosity and gas permeability, and the compressive strength of the mortar was improved by 20%. We combined the three relationship graphs. It can be analyzed that the mortar obtained when replacing natural river sand with 20% waste glass sand had a good effect on porosity, gas permeability, and compressive strength.

### 3.3. Pore Structure Analysis of Mortars

The T_2_ spectrum of mortar can be measured by a nuclear magnetic resonance (NMR) test. In mortar pore-structure research, the T_2_ spectrum is positively correlated with the amount of mortar pore water content. The T_2_ spectrum can reflect the variation of mortar-material pore volume. In the T_2_ spectrum distribution, the transverse *x*-axis represents the relaxation time, which is proportional to the pore size. The longer the relaxation time, the larger the pore size; vice versa, the smaller the pore size. The variation of the area of the T_2_ spectra corresponds to the interpretation of the pore volume. The larger the site, the larger the pore volume in the corresponding pore size range, and the smaller the pore volume in the opposite direction.

**Figure 10:**
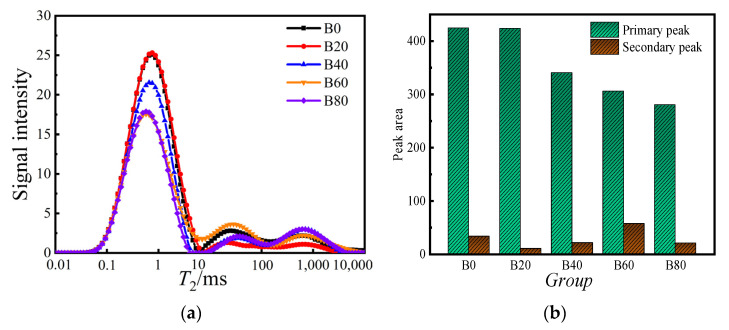
T_2_ spectrum and peak area: (**a**) T_2_ spectrum; (**b**) peak area.

The T_2_ spectrum of the NMR test is shown in Figure 10a, and it can be analyzed that the T_2_ of each doping mortar had three wave peaks. Peak area is the peak number, peak start time, peak end time, peak area, and peak ratio normalized to the primary and secondary peak area values. The primary wave peak was more significant than the secondary wave peak. The secondary wave peak represents the second and third wave peaks. The distribution of the primary wave peak occupied all three orders of magnitude. The range relaxation time corresponding to the primary wave peak was shorter, so the primary wave peak represents the change of micro and tiny pores, and the secondary wave peak represents the change of medium and large pores. As seen from the T_2_ spectrum, the primary wave peaks of the B0 and B20 specimens nearly overlapped. But the relaxation time of B20 was a little longer than that of B0, which meant that there were slightly more micropores inside the B20 specimen than inside the B0 specimen. The relaxation time between 10–100 ms and 100–1000 ms can be seen from the strength of the signal intensity. The secondary wave peaks of each doping amount of mortar were ranked from small to large as 20%, 40%, 80%, 0%, and 60%, indicating that the waste glass sand doping optimizes the internal pore structure of the mortar as a whole. The peak area of the primary and secondary peaks is shown in Figure 10b. The peak area reflected the pore volume size in the corresponding pore size range of the T_2_ spectrum. The primary peak area of 20% doped mortar was 0.21% higher than that of the control group. The primary peak areas of 40–80% doped mortar were 19.80%, 27.90%, and 34.0% lower than that of the control group. When observing the secondary peak area, the secondary peak area of 20%, 40%, and 80% mortar decreased by 70.01%, 37.32%, and 38.45%, respectively, compared with the control group.

The secondary peak area of 60% mortar was 50.02% higher than the control group. Based on a comparison of the primary and secondary peak areas of the mortar with different doping amounts of waste glass sand and the control group, it can be found that with the increase of waste glass sand doping, the mortar-pore volume size showed a general trend of decreasing and then increasing. While 20% waste glass sand doping was optimal, its mortar internal-pore structure was denser, with the least number of micropores but the least number of medium and large pores.

**Figure 11:**
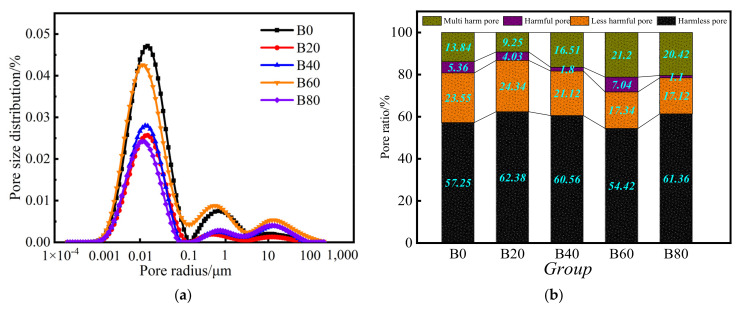
Pore size distribution and pore space distribution as a percentage: (**a**) pore size distribution; (**b**) pore space distribution as a percentage.

Figure 11a shows the pore size distribution of the mortar obtained from NMR tests. The pore size distribution was analyzed to better study the differences in the pore structure of mortars with different replacements of waste glass sand. From Figure 11a, it can be seen that the pore size pattern in the mortar was a “three peaks” distribution. The pore size range of the first peak is 0.001–0.1 μm (small pore); the pore size range of the second peak is 0.1–10 μm (medium pore), and the pore size range of the third peak is 10–100 μm (large pore). The area enclosed by the second and third peaks of the mortar at a certain replacement of waste glass sand was smaller. Thus, the pore size inside the mortar was smaller. This indicated that the fine particles in the waste glass sand formulated from waste glass particles reacted with cement hydration to form a gel that made the internal pore structure of the mortar denser. The distribution of pore percentage inside the mortar is shown in Figure 11b. The pores were classified into harmless pores (r < 0.02 μm), less-harmful pores (0.02–0.05 μm), harmful pores (0.05–0.2 μm), and multi-harmful pores (r > 0.2 μm). The pore-size-distribution diagram shows that the harmless and less-harmful pores are concentrated in the primary peak, and the harmful and multi-harmful pores are concentrated in the second and third peak intervals. From Figure 11b, it can be seen that the sum of multi-harmful and harmful pores in the pore ratio of the 20% replaced mortar was 5.92% lower than the sum of the control group, indicating that the size and number of pores inside the mortar were the smallest at 20% replacement, and the pore structure of 40% replaced mortar was the second lowest. However, with the increase of waste glass sand replacement, the number of large pores inside the mortar increased again, indicating that the optimum replacement of waste glass sand to replace natural river sand is 20% because the mortar with this replacement had the most harmless pores, the fewer harmful pores, and the densest pore structure.

### 3.4. XRD Analysis

**Figure 12:**
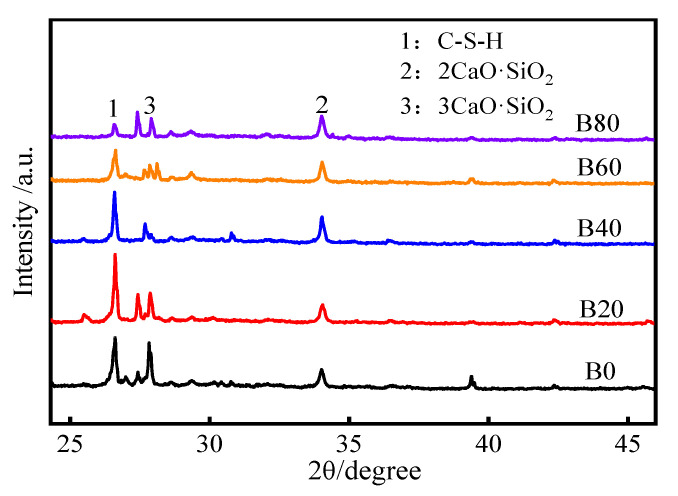
XRD pattern of mortar hydration products.

Calcium–silicate–hydrate (C–S–H) is the main product of the hydration process of silicate cement, and the source of the strength of silicate cement [41], dicalcium silicate (2CaO-SiO_2_) and tricalcium silicate (3CaO-SiO_2_) are the meta-components contained in the raw material of cement. Figure 12 shows the XRD pattern of the hydration products of mortar, from which it can be seen that the peak angle of C–S–H gel is 26.5° and that the peak angles of 2CaO-SiO_2_ and 3CaO-SiO_2_ are about 34°, and 27.85°. It can be found that the intensity of the C–S–H crystalline gel diffraction peak of group B20 is significantly higher than that of group B0. 2CaO-SiO_2_/3CaO-SiO_2_ was relatively weaker. The relative content of C–S–H gel, the main hydration product of mortar, increases at 20% replacement. Then it shows a gradual decrease with the increase of waste glass sand replacement, and 2CaO SiO_2_/3CaO-SiO_2_ decreased gradually with the replacement of waste glass sand. This indicates that, after the replacement of waste glass sand, the water absorption of waste glass sand was lower than that of natural river sand. The degree of hydration reaction was more intense at the early stage of mortar maintenance. At the same time, the volcanic ash effect of waste glass sand is more obvious in the alkaline cement paste environment. The hydration reaction of SiO_2_ in waste glass sand with alkaline material Ca(OH)_2_ produces C–S–H gel, which improves the internal compactness of mortar and improves the internal microscopic pore structure [42].

### 3.5. SEM Analysis

**Figure 13:**
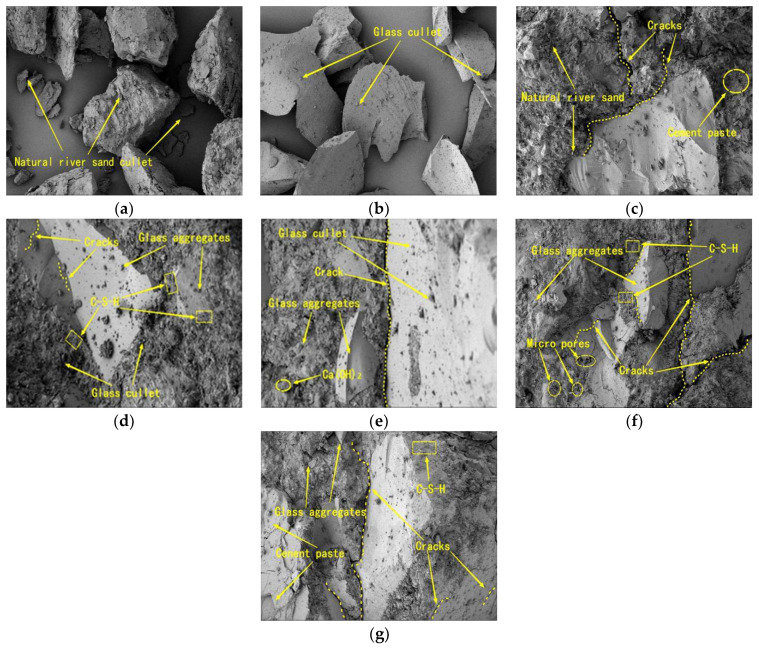
Scanning electron micrograph of mortar: (**a**) natural river sand; (**b**) waste glass sand; (**c**) B0; (**d**) B20; (**e**) B40; (**f**) B60; (**g**) B80.

Figure 13 shows the microscopic SEM images of mortar, and Figure 13a,b shows the SEM images of natural river sand and waste glass sand, respectively. The natural river sand particles are more regular in shape, wit ah rough surface texture, and the waste glass sand is irregular in shape and angular and smooth in surface texture. It can be seen that the texture of waste glass sand is denser than that of natural river sand, and both of them have broken sand on the larger grains. Since the waste glass sand was formulated according to the grain grade of natural river sand, the diameters of the two grains of sand were approximately the same. The SEM image of the control mortar is shown in Figure 13c, which shows that there were tiny gaps at the interface between the larger-grained natural river sand and the cement paste. The cracks between the smaller-grained sand and the cement paste were more obvious. The silica in the natural river sand reacted with the cement paste during the maintenance process of the mortar specimens with alkali–silica, thus creating cracks [42,43,44].

A scan of the 20% replacement is shown in Figure 13d, and it can be seen that the intersection of waste glass sand and cement paste was denser at this replacement. Almost no gap was produced. More C–S–H gel was produced at the intersection, thus inhibiting the ASR reaction and restricting the dilatation of the cement paste. A small number of microcracks were generated inside the waste glass sand. This was attributed to the extrusion during the fabrication process. The large glass particles inside the original microcracks provided favorable conditions for the formation of ASR gels. Because it was difficult to diffuse the microcracks in small spaces where dissolved silicon and sodium ions existed, leading to higher concentrations of silicon, sodium, and calcium [45]. In Figure 13e–g, the bonding between cement paste and waste glass sand decreased with the increase of waste glass sand replacement compared to the 20% replacement, due to the smoother surface texture of glass particles.

## 4. Conclusions

This study analyzed the effects of different waste-glass-sand replacements on mortar gas permeability, mechanical properties, and pore structure using a combination of macroscopic and microscopic tests. The following conclusions can be drawn:

1. When the replacement rate of waste glass sand is 20%, the permeability of mortar is the smallest, 57.4% lower than that of natural mortar. This replacement of waste glass sand and natural river sand worked together to achieve more satisfactory results. This replacement of mortar internal seepage paths was lower and narrower. The sensitivity of the mortar to changes in the confining pressure was minimized at 20%.
2. The mechanical properties of waste-glass-mixed mortar declined by 10–25% compared to the control mortar when the natural fine aggregate was partially replaced by using glass fine aggregate. However, the 20% waste glass sand replacement of natural sand increases the impermeable pore density inside the mortar, which increases the compressive strength of the mortar by 3%. To achieve a better result waste glass sand, replacement with fine aggregate by 20% can be used.
3. From the NMR test, it can be seen that, with the increase of waste glass sand replacement, the tiny internal pores and the medium and large pores of the mortar were reduced. The 20% replacement mortar had the lowest number of harmful pores; this replacement mortar’s pore structure was the densest and the best.
4. The microscopic XRD pattern and the SEM analysis of the mortar showed that more C–S–H gels were produced in the mortar with 20% replacement and that 2CaO-SiO_2_/3CaO-SiO_2_ was relatively lower. The compactness and microstructure of the mortar were optimized, which improved the waste glass sand mortar’s compressive strength and gas permeability.

This paper studies the application of waste glass as fine aggregate in cement mortar. By replacing natural mortar, the mining of natural river sand is reduced, and the resource utilization of waste glass is also achieved. This study differs from other studies in that we formulated glass sand with the same particle gradation as natural river sand. Furthermore, different substitution rates were used to improve the impact of the mortar in terms of performance and to determine the possibility of using waste glass in various forms on cement-based materials.

## Figures and Tables

**Table 1:** Mix proportion of cement mortar.

Group	Water (kg/m^3^)	Cement (kg/m^3^)	Natural Sand (kg/m^3^)	Waste Glass Sand (kg/m^3^)	Weight Replacement
B0	225	450	1350	0	0%
B20	225	450	1080	270	20%
B40	225	450	810	540	40%
B60	225	450	540	810	60%
B80	225	450	270	1080	80%

**Table 2:** Mortar dimensionless permeability fitting parameters.

Group	a	b	R^2^
B0	1.779	−0.542	0.989
B20	1.208	−0.212	0.910
B40	1.238	−0.256	0.903
B60	1.902	−0.625	0.963
B80	1.464	−0.411	0.910

## Data Availability

The data presented in this study are available on request from the corresponding author.
